# Anticipation of aversive visual stimuli lengthens perceived temporal duration

**DOI:** 10.1007/s00426-021-01559-6

**Published:** 2021-08-06

**Authors:** Ville Johannes Harjunen, Michiel Spapé, Niklas Ravaja

**Affiliations:** Department of Psychology and Logopedics, Faculty of Medicine, University of Helsinki, Haartmaninkatu 3, 00290 Helsinki, Finland

## Abstract

Subjective estimates of elapsed time are sensitive to the fluctuations in an emotional state. While it is well known that dangerous and threatening situations, such as electric shocks or loud noises, are perceived as lasting longer than safe events, it remains unclear whether anticipating a threatening event speeds up or slows down subjective time and what defines the direction of the distortion. We examined whether the anticipation of uncertain visual aversive events resulted in either underestimation or overestimation of perceived duration. The participants did a temporal bisection task, where they estimated durations of visual cues relative to previously learnt long and short standard durations. The colour of the to-be-timed visual cue signalled either a 50% or 0% probability of encountering an aversive image at the end of the interval. The cue durations were found to be overestimated due to anticipation of aversive images, even when no image was shown afterwards. Moreover, the overestimation was more pronounced in people who reported feeling more anxious while anticipating the image. These results demonstrate that anxiogenic anticipation of uncertain visual threats induce temporal overestimation, which questions a recently proposed view that temporal underestimation evoked by uncertain threats is due to anxiety.

**Supplementary Information:**

The online version contains supplementary material available at 10.1007/s00426-021-01559-6.

## Introduction

Perception of time is flexible and fluctuates alongside our internal states, sensory feedback, and situational demands (Droit-Volet & Gil, 2009; Wittmann, 2009). A good example of this flexibility can be found in introspective accounts of how time seems to slow down in threatening situations, such as a car accident or a free fall (Noyes & Kletti, 1972; Stetson et al., 2007). This often-reported experience resonates with decades of laboratory work, which shows that fear-provoking and aversive stimuli are perceived to last longer than neutral or positively valenced stimuli of the same duration (Droit-Volet et al., 2010; Tipples, 2008, 2011). For example, seeing an image depicting an aversive scene or an angry face as compared to a neutral scene or face increases the likelihood that a stimulus duration will be judged as long (Angrilli et al., 1997; Droit-Volet et al., 2004; Tipples, 2008). Similarly, expecting an aversive blast of noise or painful tactile stimulus increases perceived temporal duration (Droit-Volet et al., 2010; Fayolle et al., 2015; Ogden et al., 2015). The emotion-driven overestimation is thus not limited to direct exposure to threatening events, but already occurs when expecting something unpleasant or threatening to take place. In recent years, studying the impact of emotional anticipation on subjective time has become an increasingly important topic in time psychology because it provides an extraordinary testbed for theoretical models of time perception.

The vast majority of studies demonstrating the threat-induced temporal overestimation explain the effect in terms of the so-called pacemaker–accumulator device, which is laid out in the scalar expectancy theory (Gibbon, 1977). According to this framework, timing relies on a combination of a pacemaker emitting pulses, an accumulator that collects them, and a switch process that regulates the accumulation. Emotionally arousing events are thought to speed up the pacemaker through increased arousal, which results in faster accumulation of pulses and therefore greater estimates of elapsed time (Droit‐Volet et al., 2004, see Lake et al., 2016a, 2016b for review).

Arousal alone is unlikely to account for temporal distortions of all sorts (Lake et al., 2016a, 2016b). As an addition to the pacemaker–accumulator device, Zakay and Block (1995) introduced an attentional gate model (AGM), according to which some degree of attention needs to be allocated to the timing task for the pulses to accumulate. If the perceiver’s attention is directed away from the temporal task, then the gate to the accumulator closes and pulses are missed and this results in shorter estimates. This attentional gating is suggested to also play a role in threat-driven temporal distortions. For instance, threatening events can boost the accumulation of the pulses. This induces temporal overestimation because they capture the perceiver’s attention efficiently and therefore enable a rapid onset of the timing process (e.g. Ogden et al., 2015). Conversely, in some circumstances an expectation or direct exposure to a threat may result in reallocation of attentional resources away from the timing task towards the threat itself, which would result in underestimation of the elapsed time (Sarigiannidis et al., 2020).

Recent evidence suggests that anticipation and the perception of an aversive event will lead to temporal underestimation if the threatening event is presented as a distractor that is completely separate from the cue to be timed (Lake et al., 2016a, 2016b; Lui et al., 2011) or if its occurrence is uncertain (Sarigiannidis et al., 2020). In a recent study by Sarigiannidis et al. (2020), the participants were asked to classify durations of visual probes relative to long and short standard durations. Some of the probes were accompanied by a probabilistic cue that indicates a risk of receiving an electric shock at any point without warning. The participants were shown to systematically underestimate the durations of probes associated with the probability of a shock. The results were interpreted as reflecting the division of attentional resources between the prospective timing and preparing for the uncertain threat. This attentional division was assumed to disrupt the accumulation of temporal information. Because the occurrence of the shock was uncertain, the anticipation was supposed to elicit anxiety in the perceiver. Therefore, the authors suggested that while the acute state of fear elicited by immediate and certain threat may lead to overestimation, an anxiety that is evoked by uncertain future threats leads to an underestimation of elapsed time (Sarigiannidis et al., 2020). This argument was built on the established distinction between fear and anxiety; the former is seen as an acute negative state that is elicited by immediate or certain aversive event, whereas the latter is seen as a negative state that is elicited by the anticipation of an aversive event whose occurrence is uncertain (Davis et al., 2010).

It remains unclear if an anxiety elicited by all kinds of aversive events results in temporal underestimation. For example, in a recent study by Vallet et al. (2019), the anticipation of certain aversive versus visual stimuli resulted in an overestimation rather than underestimation of elapsed time. It is thus possible that underestimation occurs only when the anticipated event is uncertain and poses a serious threat to the perceiver's physical integrity. For instance, when anticipating an uncertain occurrence of a strong aversive event, such as an electric shock, underestimation bias might occur because the anticipation of the possible shock is prioritised and attentional resources for the timing task decrease. Anticipation of uncertain aversive visual events should therefore not result in the reallocation of attentional resources away from the temporal estimation of the cue, but may even further bias attentional gain towards the timing task.

To examine this question, we tested whether anticipation of an uncertain aversive visual stimuli results in temporal overestimation (H1) and whether the perceiver’s level of anxiety predicts the extent of this threat-driven temporal overestimation (H2). The participants completed a temporal bisection task, during which they classified durations of visual probes relative to long and short standard durations. The probe colour indicated either a 50% (threat cue) or a 0% (safety cue) probability of seeing an aversive picture of a mutilated human body in the end of the to-be-timed interval. Because the threat cue was probabilistic, there were three experimental conditions in the experiment: (1) where the aversive image was anticipated and presented at the end of the estimated time interval (threat + picture); (2) where the aversive image was anticipated but a blank screen was shown (threat + blank); and (3) where a blank screen was anticipated and presented (safe condition).

Critically, the inclusion of the threat + blank condition allowed us to disentangle the effects of the anticipation of an aversive event from those caused by the event itself. In other words, if the anticipated threatening event would have always followed the anticipatory threat cue (e.g. Droit-Volet et al., 2010; Fayolle et al., 2015), then the cue would be predictive and the threat would be certain. It would be impossible to know whether the temporal distortion was due to the anticipation of threat or due to the threat itself. Moreover, the predictive threat condition accompanied by the event would also differ from the safe condition in terms of perceptual features that could have a confounding effect on time estimation (Folta-Schoofs et al., 2014). In this study, we aimed to mitigate these confounding influences by testing the H1 by comparing the temporal estimates in the safe condition to those in the threat + blank condition. Consequently, there were no significant perceptual differences between the two conditions other than the cue colour, which was counterbalanced between the participants.

Note that in contrast to some previous studies (Sarigiannidis et al., 2020; Vallet et al., 2019), in the current study, the aversive events immediately followed the to-be-timed interval, rather than being shown after the explicit (long/short) response. The alternative approach of presenting the aversive picture after the response was deemed to be suboptimal because it decreases the uncertainty of the event’s occurrence in time and increases the participant’s control over the moment of the event as always following their response. In other words, in the current study both the occurrence and the time point at which the event possibly occurred were unpredictable (because of the varied cue duration), which generates the maximum amount of uncertainty.

Finally, we examined whether the level of anxiety reported during anticipation was really associated with the extent of temporal distortion, as suggested but not directly tested by Sarigiannidis et al. (2020). To answer this question, we used multilevel linear modelling to test H2; according to which people reporting higher levels of anxiety in the anticipation period would exhibit a stronger threat-driven temporal overestimation than those reporting lower levels of anxiety.

## Methods

### Participants

Our sample consisted of 42 healthy adult volunteers who were recruited via the University of Helsinki student organisation email lists. Of these, 30 identified their gender as female, 10 as male, and 2 as non-binary with an average age of 29.00 years (SD = 8.61). The required sample size was estimated based on a hypothesised difference between the threat + blank and safe condition. Prior studies indicated a large effect of threat anticipation on timing—the effect size (Cohen’s d) varies from *d* = 1.89 (Fayolle et al., 2015) to 0.76 (Sarigiannidis et al., 2020, Study 1). Power calculation (https://jakewestfall.shinyapps.io/pangea/) informed that finding an effect of *d* = 0.76 with 80% statistical power would require a sample of 38 participants. Consequently, a sample of 42 participants was collected. No exclusion criteria were used for the participation but those who were especially sensitive to disgust-provoking visual content were not encouraged to participate in the study. The data of two of the participants were removed due to monotonic response style (i.e. consistently estimating all durations as long or short). Therefore, the final sample consisted of 40 participants [28 females, 10 males, and 2 non-binary with an average age of 29.16 years (SD = 8.82)]. The study was performed in accordance with the ethical standards as laid down in the 1964 Declaration of Helsinki and was approved by the University of Helsinki research ethics committee. The participants were paid 7 euros as compensation for their time.

### Procedure

The data were collected remotely using E-prime Go, which is a recent and self-contained run-time version of the E-Prime 3.0 software (Psychology Software Tools, Pittsburgh, PA, USA). The experiment was hosted on an online server, which was run locally on the participant’s own Windows PC by downloading the experiment via a link attached to the invitation letter. Before starting the task, the participants were informed about the experiment and their right to withdraw from the study at any point. They then gave their informed consent through the experiment system.

#### Conditioning task

After giving their informed consent, the participants went through a short conditioning task to learn to associate a certain coloured (e.g. pink) cross to a subsequent aversive picture and another coloured cross (e.g. blue) to the presentation of a blank grey screen. The aversive pictures presented in this task were randomly selected from a pool of 29 mutilation pictures^1^ that were drawn from the International Affective Picture System (IAPS, Lang et al., 1997). The conditioning task consisted of eight trials, four with a predictive threat cue (e.g. pink cross) and subsequent aversive picture, and four trials with a predictive safety cue (e.g. blue cross) and a subsequent blank screen. Each trial began with a fixation dot of 900–1400 ms (randomised), followed by a framed cross shown for 3000 ms. Depending on the colour of the cross, a picture of a mutilated body (threat trial) or a blank screen (safe trial) followed and was in view for 500 ms (see Supplementary Fig. 1 for the trial structure). The participants were not informed that the threat cue was 100% predictive of the aversive events. Instead, to increase uncertainty and evoke anxiety, they were told that the threat cue indicated that an aversive event could be shown afterwards. The safety cue was informed to be predictive and indicated that no aversive picture would be shown.

#### Bisection training

Next, the subject learned to distinguish between a short-lasting duration of 800 ms and a long-lasting duration of 2600 ms. Each of these training trials was initiated with a 900–1400 ms (randomised) presentation of fixation dot, after which the timing cue (framed cross) was shown. The colour of the cross indicated safe condition and no picture was shown afterwards throughout the training. The training consisted of 14 trials where the cue duration was varied randomly between short (800 ms) and long (2600 ms) duration. In the end of each trial, the participants indicated using the “z” and “m” letter keys of their keyboard whether the cue duration was long or short. Feedback about whether their response was correct was shown afterwards (see Supplementary Fig. 1 for the trial structure).

#### Anticipatory bisection task

**Fig. 1:**
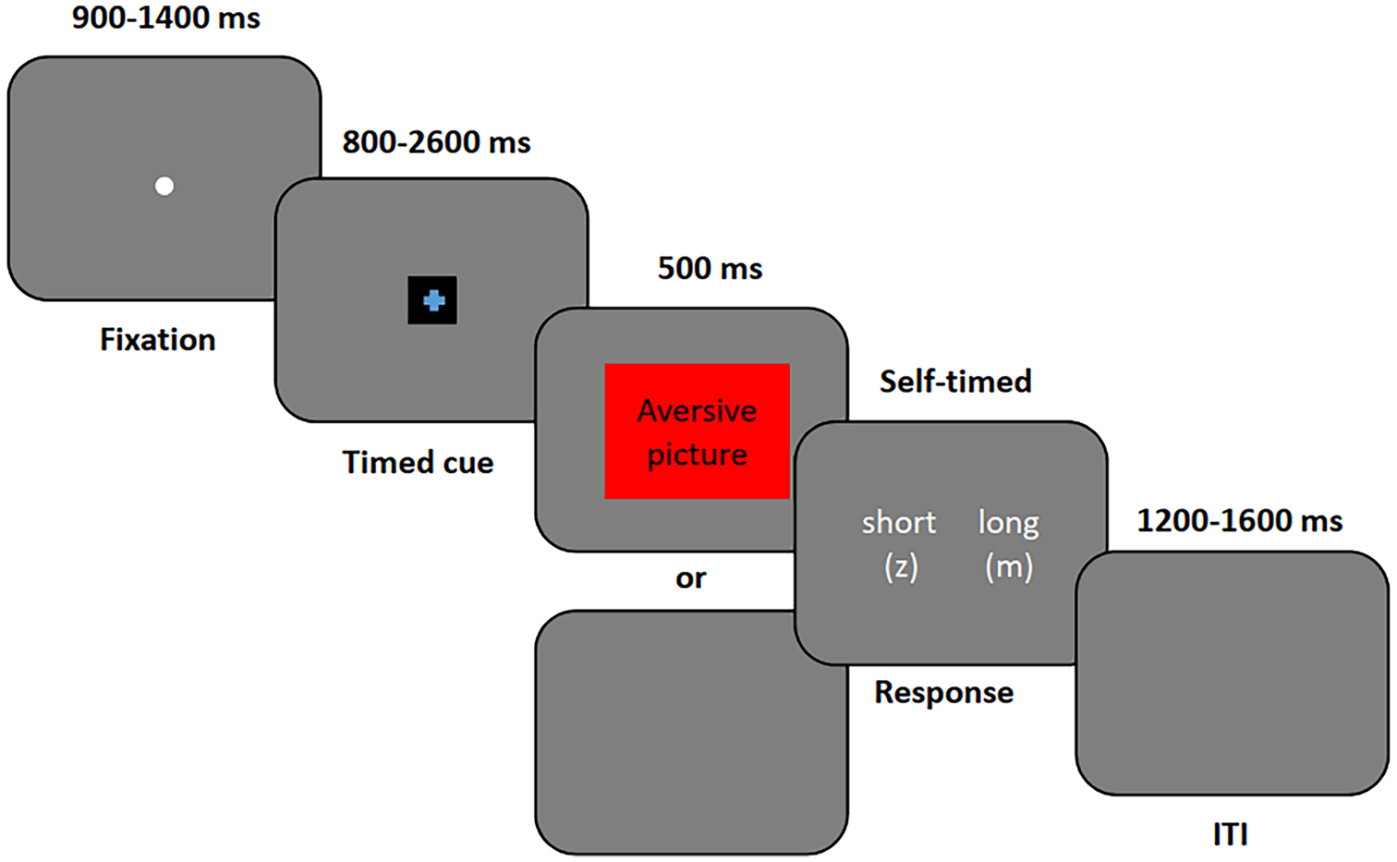
Trial procedure with timing information

The trial structure of the bisection task with anticipatory cues is illustrated in Fig. 1. Each trial started with a 900–1400 ms (randomised) fixation, which was followed by a timing cue of variable duration (800, 1100, 1400, 1700, 2000, 2300, or 2600 ms). The cue colour referred to the condition indicating either 50% (threat cue) or 0% (safety cue) probability of a subsequent aversive picture appearing on the screen. Following the variable duration, either the aversive image, or the blank screen was presented for 500 ms (depending on the condition). The participants then indicated using the z and m keys (key-response mapping counterbalanced between participants) how the target stimulus compared to the short and long comparison intervals that were introduced during the training. Upon response, a 1200–1600 ms blank inter-trial interval (ITI) followed. There were 448 trials in the task that were divided into four blocks of 112 trials in each. In 50% of the trials (i.e. 224), the colour of the cross indicated zero probability of aversive image and a blank screen was shown (safe trials). The participants were informed that the safe trials had 100% cue-event contingency, which means that a blank screen would always follow a safety cue. In the other 224 trials, the colour signalled a probability of an aversive image but only in 112 (50%) of these trials was such an image actually shown. In these threat trials, the cue–event contingency was also told to be probabilistic, but no exact probabilities of event occurrences were revealed to the participants. Consequently, for each duration category, there were 32 safe trials, 16 threat + picture trials, and 16 threat + blank trials.

### Stimuli

Due to the remote data collection approach, we had no control over the display technology and response devices that were used by the participants. However, the experiment advisor reports (EARs) that were generated by E-prime Go in each session were analysed to ensure that the participants’ devices met the minimal requirements of E-Prime 3.0.

#### Visual stimuli

The fixation was a white dot presented on a grey background in the middle of the screen. The to-be-timed cue consisted of a black square (28 × 28 pixels) and a coloured fixation cross (i.e. plus sign with font size 30) within it. The cross colours were equiluminant light pink (HSV coordinates: 308, 75, 100) and light blue (HSV: 211, 75, 100). The intensity and saturation were held constant and only the hue was varied. The aversive visual stimuli consisted of 29 mutilation pictures^2^ that were drawn from the International Affective Picture System (IAPS, Lang et al., 1997). Only pictures depicting severely injured bodies were included. Based on a technical report of the IAPS (Lang et al., 2008), these pictures are rated as negatively valenced (*M* = 1.83, SD = 0.44) and highly arousing (*M* = 6.47, SD = 0.59). The blank screen shown in the safe condition was grey (RGB: 127, 127, 127) with a hue of 160, luminance of 120 and zero saturation.

### Measures

#### Self-reports

State of anxiety was measured in each trial of the conditioning task. The participants were asked to evaluate their anxiety level when anticipating an upcoming image using a single item (“How nervous/worried/anxious did you feel when looking at the cross”). The participants were unaware that the probability of seeing an aversive picture after each threat cue was actually 100 percent in the conditioning task. The supposed uncertainty of the picture occurrence was expected to elicit anxiety. The responses were given on a five-point Likert scale (1: not at all nervous/worried/anxious; 5 extremely nervous/worried/anxious adjectives varied sequentially between the trials). The questionnaire represented an adapted form of the State-Trait Anxiety Inventory (STAI; Spielberger et al., 1983). The participants also evaluated the aversiveness of mutilation images during the conditioning task. Here, a single item (“How unpleasant was the image?”) with a five-point Likert scale (1: not at all unpleasant; 5: extremely unpleasant) was used.

### Duration estimation

Data from the bisection task were first presented as probabilities in which each cue duration (800, 1100, 1400, 1700, 2000, 2300, or 2600 ms) was estimated as long. This was done separately for each of the three cue conditions (safe, threat + blank, and threat + picture). Assuming that the participants had learned to discriminate the long and short duration, the probability of long response in the shortest duration (800 ms) was predicted to be close to zero, whereas the probability of the longest one (2600 ms) was assumed to approach 100%. The probabilities of long responses across the different cue durations are known to follow a sigmoidal S-shaped response function. The parameter of interest derived from this response function was the bisection point (BP, also known as point of subjective equality) that represents duration value at which the long and short response become equally likely (Prins & Kingdom, 2016). This provides a measure of the perceived duration of the cue durations and allows examination of distortions in duration estimation. A leftward shift of the psychometric response function results in a smaller BP value (i.e. the 50% threshold is reached earlier), which indicates overestimation of perceived duration.

### Data analysis

The experiment completion time and responses of each participant were visually inspected to determine attentiveness of the participants. Then, the aversiveness ratings of the remaining 40 participants were examined to ensure that they perceived the images as unpleasant. A paired samples *t* test was used to compare anxiety ratings in the safe cue and threat cue condition to find whether anticipating an aversive image induced anxiety in the participants. For the duration estimation data, bisection task responses with too fast or slow reaction times were first removed from the data before the analysis using the median absolute deviation (MAD) method with a conservative threshold value of 3 for defining outliers (Leys et al., 2013). To examine the duration estimation in the three cue conditions, the BP values for each participant and each condition were determined by fitting a sigmoidal S-shape mathematical function^3^ to the cleaned binary trial-level response data using long responses as the outcome and cue duration as the predictor. Estimation by direct maximisation of the likelihood (Prins & Kingdom, 2016) was used as implemented in the quickpsy R package (Linares & López-Moliner, 2016; version: 0.1.5.1). The BP values for each participant and for each of the three cue conditions were then extracted from the fitted functions.

To examine the differences between the average BP values of the three conditions, a multilevel linear model (MLM) with cue condition as a factor was calculated using the lmer function for R utilising the restricted maximum likelihood (REML) estimation method. The modelling was conducted in a stepwise manner by testing the fixed effect of cue condition (H1), followed by addition of the anxiety tendency to the model as person-level covariate, and finally the interaction effect between the two variables. The interaction effect was examined to see whether people with higher anxiety exhibited stronger differences in their bisection points between the threat + blank and safe condition than those with lower anxiety (H2). Although the threat + picture condition was also examined, there were no specific hypotheses regarding duration estimation in this condition because the picture presentation itself could have influenced the estimation. In all of the models, the intercept was defined as a random effect. No random slopes were included. The omnibus test of the fixed effects utilised type-III analysis of variance with Satterthwaite's method.

## Results

### Inspection of the participants’ engagement

The experiment completion time and responses of each participant were inspected to determine the attentiveness of the participants. The average completion time of the experiment was 58.54 min (SD = 24.61). The responses of each participant revealed that two participants had a monotonic response style (i.e. selecting a long response in all trials). The data were removed, resulting in a final sample size of 40 individuals. The responses of those completing the task the slowest (2:54 h) and fastest (43 min) were also inspected with care but no evidence of low engagement was found and the psychometric response functions of these individuals were similar to those with average completion time.

### Aversiveness and anxiety ratings

Across the conditioning task, participants rated the pictures as moderately aversive (*M* = 3.11, SD = 0.87) on a five-point Likert scale. They also felt significantly more anxious in the threat condition when anticipating an aversive picture (*M* = 2.43, SD = 0.87) than in the safe condition, where they knew that no aversive picture was going to be presented (*M* = 1.31, SD = 0.42), *t*(39) = 8.29, *p* < 0.001.

### Duration estimation

**Fig. 2:**
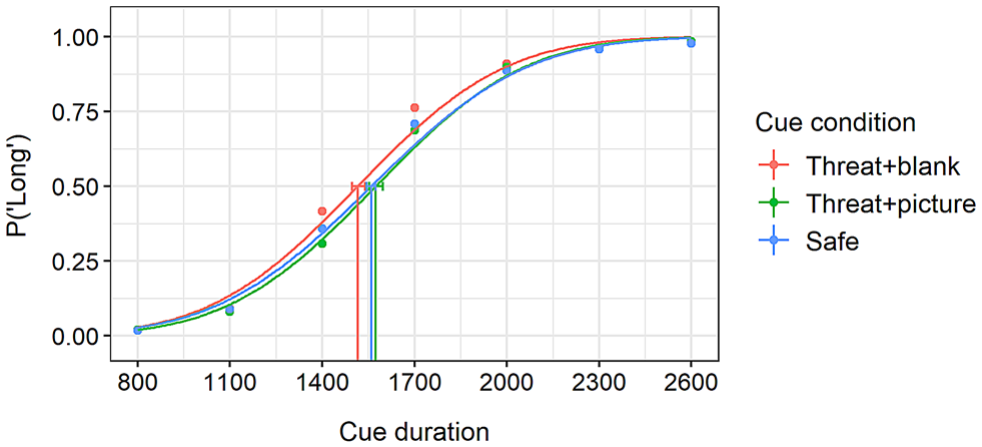
Probability of cues perceived as long in the safe, threat + picture, and threat + blank condition as a function of cue duration. The coloured dots represent average probabilities of long responses in each cue condition and cue duration. The three curves present psychometric functions of the three cue conditions fitted to the binary bisection task responses. The error bars on the curves refer to 95% confidence intervals obtained with a parametric bootstrap method

Figure 2 shows the probability of responding long as a function of seven comparison durations and three cue conditions: threat + picture, threat + blank, and safe. As can be seen, the bisection point of the threat + blank condition was earlier in time and the probability of responding long was therefore higher in the threat + blank as compared to the safe condition or the threat + picture condition. This suggests that anticipating an aversive picture results in overestimation of temporal durations but only when no aversive picture was shown at the end. Examining the psychometric functions separately for each participant revealed considerable variation between participants in the threat + picture condition. Some showed a strong overestimation effect similar to the one observed in the threat + blank condition, whereas others showed an underestimation when compared to the safe control condition. This interindividual variation between overestimation and underestimation was so large that no group-level difference was found between the safe and threat + picture condition.

MLM with cue condition as a factor was calculated to statistically test the difference of average BP values among the threat + picture, threat + blank, and the safe condition. The MLM results are summarised in Table 1, which shows estimates (i.e. regression coefficients), standard errors, and significance test results of three nested models. The first model (M1) included only the fixed effect of cue condition predicting BP. The omnibus test of the factor was statistically significant, *F*(2,78) = 4.65, *p* = 0.012. Estimated coefficients of the Table 1 (M1) reveal that the bisection point values are significantly smaller in the threat + blank (*M* = 1504.85 ms, SE = 28.75) than in the safe [*M* = 1555.69 ms, SE = 28.75, *t*(39) = 3.01, *p* = 0.005, *d* = 0.96], or threat + picture condition [*M* = 1570.63.69 ms, SE = 28.75, *t*(39) = 2.63, *p* = 0.012, *d* = 0.84]. The difference between threat + picture and safe was not significant (see M1). The results support H1, which states that anticipation of uncertain aversive visual event leads to temporal overestimation.

**Table 1:** Multilevel linear models predicting temporal bisection point with cue condition and anxiety tendency

Predictors	M1: cue			M2: anxiety			M3: cue*anxiety		
	Estimates	SE	*p*	Estimates	SE	*p*	Estimates	SE	*p*
Intercept	1555.69	28.75	< 0.001	1555.69	27.44	< 0.001	1555.69	27.49	< 0.001
Threat + picture vs. safe	14.93	22.63	0.509	14.93	22.63	0.509	14.93	22.83	0.513
Threat + blank vs. safe	− 50.84	22.63	0.025	− 50.84	22.63	0.025	− 50.84	22.83	0.026
Anxiety				− 59.07	24.23	0.015	− 57.56	27.61	0.037
Threat + picture*Anxiety							6.80	22.92	0.767
Threat + blank*Anxiety							− 11.34	22.92	0.621
*σ*^2^_Within subject_	10,241.47			10,241.47			10,423.30		
τ_00 between subjects_	22,818.17			19,866.29			19,805.68		
ICC	0.69			0.66			0.79		
Marginal *R*^2^	0.024			0.125			0.126		

*N* = 40 for all models. The total number of observations was 120. Marginal *R*^2^ refers to the amount of variance explained by the fixed effects (upper part of the table). The intraclass correlation (ICC) indicates the ratio of variance on the two levels of analysis (within-subject *σ*^2^ level and between-subjects *τ*_00_ level). *p < 0.05, **p < 0.01, ***p < 0.001

Next, we examined whether the participants' anxiety ratings in the conditioning task explained the variation in the timing behaviour and the temporal overestimation effect caused by anticipating threat. For this purpose, average anxiety scores from the anticipation period of the conditioning task were calculated for each participant, Z-score standardised, and entered as a person-level covariate into the MLM. The effect of anxiety tendency (Table 1, M2) resulted in a statistically significant omnibus test result, *F*(1, 38) = 5.95, *p* = 0.020. Inspection of the model estimates revealed that those who reported higher anxiety had earlier bisection point values, and therefore a stronger tendency to overestimate the cue duration (see Table 1, M2).

**Fig. 3:**
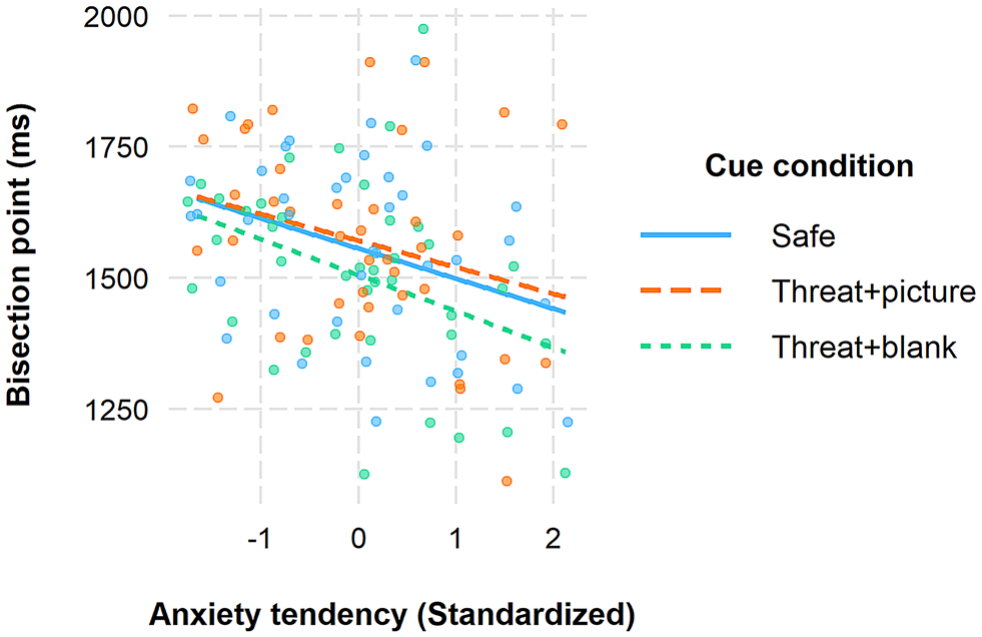
Linear relationship between anxiety tendency and estimated bisection point plotted separately for the three cue conditions. The lines represent regression slopes calculated using the MLM estimates of the fixed effects. Anxiety tendency was standardised around mean

When examining the interaction between the two variables in the next step (M3), no sign of interaction effect was found, *F*(2, 76) = 0.32, *p* = 0.727. The overestimation tendency of more anxious individuals was thus not limited to the threat + blank condition but also occurred in the safe cue condition. The simple slopes of the model estimates presented in Fig. 3 support this interpretation. The negative relation between anxiety and bisection data was statistically significant in the threat + blank (*r* = − 0.38, *p* < 0.001) and in the safe condition (*r* = -0.33, *p* = 0.035), but not in the threat + picture condition (*r* = − 0.27, *p* = 0.094).

Examination of the random effects of the models (lower part of Table 1) revealed that about 70–80% (M1: ICC = 0.69; M2: ICC = 0.66; M3: ICC = 0.79) of the variation in bisection points was accounted for by differences between people leaving about 20–30% to be accounted for by situational factors. Marginal *R*^2^ of the models implied that the variance explained by the fixed effects increased from 2.4% (M1) to 13% (M2 and M3) after including the anxiety tendency to the model.

## Discussion

In this study, we examined whether anticipation of aversive visual stimuli distorts estimation of temporal durations and whether this distortion is due to anxiety elicited by the anticipated threat. We found that anticipating an aversive visual stimulus leads to an overestimation of the elapsed time and that those reporting higher levels of anxiety when expecting the picture exhibited a generally greater temporal overestimation. In this section, we will discuss the findings in detail and elaborate upon their relationship to previous research on the topic.

In line with previous studies linking fear-provoking situations to temporal overestimation, we found that when expecting an aversive picture to be presented on screen, the duration of the anticipatory cue was perceived to be longer than when expecting a blank screen to be presented. This finding is consistent with previous laboratory studies where both direct exposure to aversive event (i.e. pictures, sounds, and somatosensory stimuli) and expecting an aversive event have been shown to slow down subjective time (e.g. Angrilli et al., 1997; Tipples, 2008; Fayolle et al., 2015; Ogden et al., 2015; Droit-Volet et al., 2010). The effect has been attributed to an increase in arousal accelerating an internal pacemaker-like process and leading to higher amount of temporal evidence or pulses being accumulated (Droit‐Volet et al., 2004).

Recent studies have demonstrated caveats to this arousal-based account showing evidence that direct exposure or expectation of emotional events sometimes shortens perceived duration (Lake et al., 2017; Sarigiannidis et al., 2020). This threat-driven underestimation effect has been suggested to arise from a reallocation of attention, resulting in less pulses being accumulated and duration being estimated as shorter (Sarigiannidis et al., 2020). We tested the generalisability of this model to other types of threat by examining whether duration estimation is distorted when anticipating a visual threat that is uncertain, aversive, but not seen as dangerous as an electric shock. Based on our finding that anticipating an uncertain visual threat elicits temporal overestimation rather than underestimation, we argue that the direction of temporal distortion depends both on uncertainty and on the level of danger associated with the anticipated threat. A competing explanation could, of course, be that the sensory modality and spatial distance between the timing cue and the threat defines the shift. In other words, expecting an aversive picture could boost attention to the visually presented timing task, whereas anticipating an electric shock to be administered to one's arm could direct attention away from the visual timing task (Sarigiannidis et al., 2020). However, this is unlikely to be the case because threat resulted in temporal overestimation in previous timing studies on emotional anticipation that used auditory or tactile threats and a visual timing task (Fayolle et al., 2015; Ogden et al., 2015; Droit-Volet et al., 2010). Therefore, the combination of uncertainty and perceived dangerousness of a threatening event better explains the shift between temporal overestimation and underestimation than differences in modality or spatial properties between the cues.

By measuring individual differences in anxiety during threat anticipation, we showed that higher anxiety scores were associated with the time intervals being seen as longer. This finding demonstrates that the link between anxiety and duration estimation is not as straightforward as suggested previously (Sarigiannidis et al., 2020). In other words, anxiety evoked by uncertain visual threat does not lead to temporal underestimation, but is associated with overestimation of elapsed time. Of course, the anxiety measure used in the current study also had its limits. Anxiety was measured four times during the conditioning task but not during the actual bisection task. Therefore, the acquired ratings might be more informative about the participant’s general tendency to feel anxious while waiting an aversive visual stimulus rather than about their acute level of anxiety during the bisection task.

In addition to more comprehensive measures of state anxiety, we still need direct evidence of the shift from temporal overestimation to underestimation to conclude that predictability and severity of the anticipated threat define the direction of temporal distortion. Consequently, one could systematically manipulate the uncertainty and perceived dangerousness of an anticipated threat within the same timing paradigm. Moreover, while the temporal overestimation effect has been demonstrated with threats coming from different sensory modalities, future research could further examine the effects of different threat modalities on emotional anticipation and duration perception. Another limitation that could be taken into account in future research is that in the current study the anticipation of threat was only contrasted to a condition in which no picture was shown. While the control condition with blank screen made the threat + blank and the safe condition comparable in terms of low-level perceptual features, one could argue that the difference in timing reflected the mere effect of expectation. Indeed, expecting even a soft tone leads to longer estimated time than expecting nothing to happen (Droit-Volet et al., 2010). However, it is unlikely that our observation of temporal overestimation was due to the expectation per se because anticipating an aversive noise results in much larger overestimation than anticipating a soft tone (Droit-Volet et al., 2010). Furthermore, in the current study, higher anxiety predicted higher temporal overestimation.

To conclude, in contrast to recent studies which suggest that anxiety elicited by uncertain threat leads to underestimation of elapsed time, we demonstrated that anticipating unpredictable aversive visual threat lengthens rather than shortens perceived duration. A further finding that anxiety during picture anticipation was associated with longer perceived durations leads us to conclude that there is no direct association between anxiogenic situations and temporal underestimation but that the direction of temporal distortion may be defined by the combination of perceived predictability and type of the threatening event.

## Supplementary Information

Below is the link to the electronic supplementary material.

Supplementary file1 (PNG 27 kb)

## Data Availability

Data and analysis script are accessible at the Open Science Framework (https://osf.io/frab3/).

## Footnotes

1 IAPS images: 3000, 3010, 3015, 3016, 3019, 3051, 3053, 3059, 3060, 3061, 3062, 3063, 3064, 3068, 3069, 3071, 3080, 3100, 3101, 3102, 3120, 3130, 3131, 3140, 3150, 3160, 3168, 3170, 3213.

2 IAPS images: 3000, 3010, 3015, 3016, 3019, 3051, 3053, 3059, 3060, 3061, 3062, 3063, 3064, 3068, 3069, 3071, 3080, 3100, 3101, 3102, 3120, 3130, 3131, 3140, 3150, 3160, 3168, 3170, 3213.

3 The function followed the form ψ(x) = γ + (1—γ—λ) * fun(x), where γ was the guess rate, λ was the lapse rate and fun was a sigmoidal-shape function with asymptotes at 0 and 1.
