# Supplementary figures and images for: Anticipation of aversive visual stimuli lengthens perceived temporal duration
Source: Psychol Res. 2021 Aug 6;86(4):1230–8. doi: 10.1007/s00426-021-01559-6 (PMC9090676; doi:10.1007/s00426-021-01559-6)

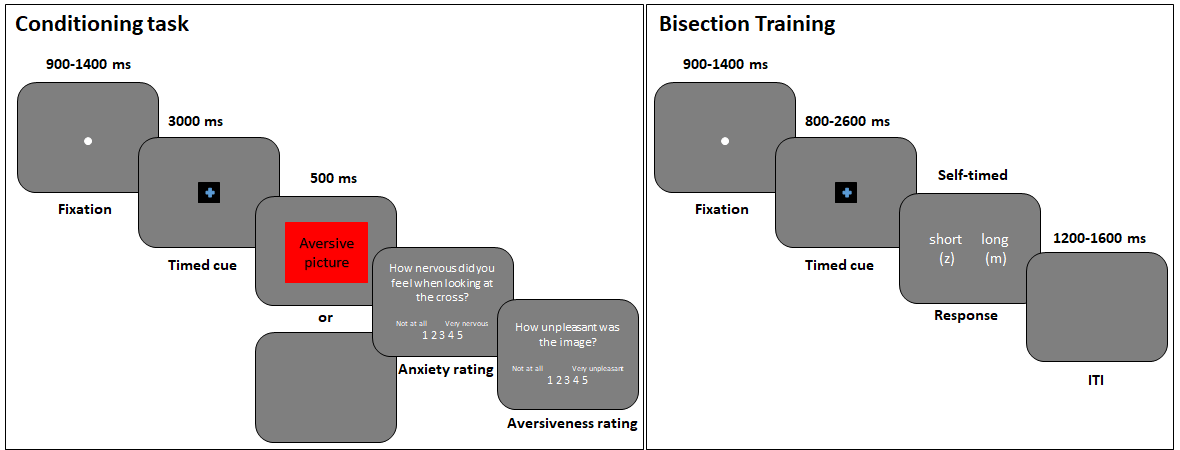

Supplement: Supplementary file 1 — Supplementary file1 (PNG 27 kb) [file 426_2021_1559_MOESM1_ESM.png]
